# Digitally-enhanced medication-assisted treatment for opioid use disorder: acceptability, engagement and treatment retention

**DOI:** 10.3389/fpsyt.2025.1581298

**Published:** 2025-10-02

**Authors:** Jorge E. Palacios, Robert Sherrick, Sara Lorenzen, Jenna Tregarthen

**Affiliations:** ^1^ Bright Therapeutics, San Francisco, CA, United States; ^2^ Community Medical Services, Scottsdale, AZ, United States

**Keywords:** opioid use disorder, digital health, retention, addiction, digital app

## Abstract

**Background:**

Opioid use disorder (OUD) is a persistent public health crisis, with medication for opioid use disorder (MOUD) significantly reducing overdose risk and improving outcomes. However, treatment adherence and retention remain critical challenges. Digital health tools offer promising solutions, yet their integration into MOUD programs has been limited. This study evaluates the implementation of *Recovery Connect*, a mobile application designed to enhance engagement, communication, and adherence in MOUD treatment.

**Methods:**

This observational study assessed the adoption and impact of *Recovery Connect* across 53 opioid treatment clinics over 12 months. A total of 11,495 patients and 302 mental health professionals (MHPs) engaged with the app, which facilitated real-time patient-clinician communication, self-monitoring, and access to evidence-based resources. Patient acceptance, engagement patterns, and clinical outcomes, particularly 30-day retention, were analyzed using survey responses, app usage metrics, and historical retention data.

**Results:**

Patient and clinician acceptance of the app was high, with 83.7% of patients who completed baseline acceptance survey expressing intent to use it. Early engagement—particularly self-monitoring and clinician-initiated messaging—was significantly associated with increased retention. Compared to historical data, 30-day retention improved by 12.2% across all clinics and by 13.2% in Arizona-based clinics. Patients with higher app engagement and more frequent clinician interactions had significantly greater odds of remaining in treatment.

**Conclusion:**

Digital tools such as *Recovery Connect* show promise in addressing key barriers to MOUD retention by enhancing patient accountability, self-monitoring, and clinician-patient communication. These findings support the integration of digital interventions into standard MOUD care, with future research needed to assess long-term retention and scalability.

## 1 Introduction

The opioid crisis continues to be a significant public health challenge, with opioid use disorder (OUD) affecting millions of individuals globally. In the United States alone, opioid overdoses resulted in nearly 80,000 deaths in 2023, highlighting the severity of the epidemic (1). Medication for Opioid Use Disorder (MOUD) has emerged as a highly effective approach in managing OUD (2), combining medications like methadone, buprenorphine, or naltrexone with counseling and behavioral therapies. Studies have shown that MOUD can reduce opioid use and overdose deaths by more than 50% (3). Additionally, MOUD has been associated with improved social functioning and reduced transmission of infectious diseases (4).

Despite its effectiveness, the implementation and adherence to MOUD protocols face numerous barriers. Ensuring that patients take their daily doses and remain accountable to their treatment plans is critical for the success of MOUD programs (5). Furthermore, Hser et al. (6) found that long-term retention in MOUD is essential for sustained recovery, yet dropout rates remain high, as studies continue to report factors such as availability of treatment, personalization, and motivation as key to retention for individuals with OUD (7). Additionally, a recent study using machine learning models in over 2.3 million treatment episodes found that systemic factors, including treatment setting, geographic region, referral source and primary source of payment were more predictive of dropout than individual factors such as age, ethnicity and education (8).

To scale up MOUD and support clinicians and clinics in helping patients maintain their treatment plans, it is crucial to address these barriers. Current treatments are effective when patients adequately participate, but they often lack the necessary infrastructure to ensure patients remain accountable outside of clinic visits. Technology can play a transformative role in facilitating the entire treatment process and removing barriers to participation. For example, Mobile apps provide flexible, 24/7 access to evidence-based resources, counsellor connection, and medication management. This flexibility is particularly critical in rural and underserved areas, where treatment facilities may be scarce. For instance, mobile health (mHealth) applications (apps) like reSET-O, approved by the FDA, have demonstrated improved adherence to buprenorphine and reduced dropout rates by delivering cognitive-behavioral therapy (CBT) modules and connecting patients with clinical support remotely (9). The anonymity offered by digital platforms also reduces the stigma associated with OUD treatment. Patients can access resources privately, without fear of judgment or discrimination. A systematic review by Lin et al. (10) found that participants using digital tools reported decreased stigma and increased self-efficacy in managing their recovery.

Several technologies and apps have attempted to support clinicians in MOUD clinics. Apps like reSET-O and DynamiCare provide reminders for medication intake, telehealth options for counseling, and platforms for tracking patient progress (11). While digital tools such as these have shown promise, their adoption has been limited, and they have not yet reached a scale sufficient to meet the needs of many individuals requiring care. A cross-sectional study analyzing data from a national survey of 276 U.S. healthcare organizations with accountable care organization contracts found that only 34% utilized at least one category of digital health technology for OUD treatment (12). This indicates that a significant majority of organizations have not integrated digital tools into their OUD treatment protocols. Integrating digital tools into existing healthcare provider networks will be critical for scaling their impact (3). Additionally, gaps remain in providing a comprehensive solution that meaningfully integrates into all aspects of MOUD care delivery, including real-time adaptive cognitive-behavioral monitoring, connection and ease of communication between patients and clinicians, and data analytics to inform treatment adjustments. A study by Lin et al. (13) emphasized the need for integrated telemedicine solutions that can offer continuous support and adapt to individual patient needs. Indeed, SAMHSA recently advised that clinical integration is key to achieving optimal outcomes with these tools (14).

In response to these challenges, this paper explores the results of implementing a comprehensive mobile app designed to integrate into MOUD clinical treatment and support both patients and clinicians throughout the care journey. The app aims to enhance treatment adherence, facilitate better communication, and provide real-time monitoring and support. By leveraging technology, the app seeks to remove barriers to patient care participation, streamline clinical workflows, and ultimately improve patient outcomes. This study will not only assess the acceptability of the app and feasibility of scaling-up its adoption within the context of a large network of MOUD treatment centers but will also evaluate user satisfaction and early outcome data to demonstrate its potential impact on improving patient retention in MOUD programs.

## 2 Materials and methods

### 2.1 Study design

This study employed an observational design to evaluate the implementation and impact of the “Recovery Connect” mobile application within an Opioid Treatment Program (OTP) providing MOUD. The observational period spanned twelve months, from July 2023 to June 2024, across 53 clinics operated by Community Medical Services (CMS). The primary aim was to assess the acceptability, engagement levels, and clinical outcomes associated with the use of the Recovery Connect app as an adjunctive tool in MOUD programs during this rollout period. The use of an observational design with historical comparisons allowed us to capture real-world adoption, acceptability, and early outcome signals across a large and diverse network of clinics. The absence of a matched control group reflects the pragmatic focus of this study on feasibility rather than causal inference.

### 2.2 Setting

The study was conducted at Community Medical Services (CMS), a large network of OTPs across numerous states in the US, specializing in providing MOUD. CMS clinics offer evidence-based pharmacological interventions, including the FDA-approved medications buprenorphine, methadone, and naltrexone, combined with counseling, case management, and other supportive services tailored to address the complex needs of individuals with OUD. New intake patients undergo an initial assessment to determine an appropriate treatment plan. Medication is prescribed and monitored by licensed medical providers, with regular visits scheduled to evaluate progress and adjust treatment as needed. Counseling and group therapy sessions are incorporated into the comprehensive care model. The clinics aim to create a judgment-free, patient-centered environment to facilitate engagement in treatment. Demographic and clinical data, including patient characteristics, substance use history, treatment engagement, and clinical outcomes, are routinely collected as part of standard clinical practice at CMS clinics.

The extensive network of CMS outpatient clinics, with established MOUD programs and data collection procedures, provided a relevant real-world setting to conduct research on the implementation and effectiveness of integrating digital health tools into OUD treatment. For the purposes of this study, we included 53 clinics which had implemented the Recovery Connect app during this extended rollout period. These clinics were situated across 9 states and distributed as follows: Arizona (24 clinics), Colorado (4 clinics), Indiana (1 clinic), Michigan (2 clinics), Minnesota (2 clinics), Ohio (9 clinics), Oregon (1 clinic), Texas (5 clinics) and Wisconsin (5 clinics).

### 2.3 Participants

The study involved two main groups of participants:

#### 2.3.1 Mental health professionals

A total of 302 MHPs at CMS clinics who are responsible for interacting with patients through the app were also a part of the study. Each clinic received uniform training to ensure proficiency in the app’s use. This training began with a mandatory introductory session covering key aspects of the app and methods for MHPs to link and connect with both new and existing patients. Continuous training opportunities were also offered for those seeking further guidance or having additional questions.

MHPs participating in the training come from diverse educational backgrounds, ranging from high school diplomas to advanced degrees in social work and counseling. Notably, some MHPs had no prior experience in counseling or substance use treatment. The training was carefully crafted to address these varying levels of prior experience, aiming to equip every MHP with the skills needed to use the app effectively in clinical settings.

The training protocol included live virtual sessions, lasting on average 2 hours, at each clinic, conducted as part of a phased, state-by-state rollout. These sessions were mandatory for all MHPs, managers, and clinical leaders, who also received an additional hour of training. Follow-up support of 1 hour for each clinic was provided virtually two weeks after the initial rollout and continued as needed for individual MHPs who requested it. Additionally, a 30 minute, bi-monthly training session, ‘Optimizing Recovery Connect,’ was introduced to provide deeper clinical insight into the Recovery Connect tool for MHPs, focusing on feature updates and aligning activities with current clinical topics for each week.

Additional 1-hour virtual training sessions were regularly scheduled for other clinic staff, such as front desk personnel, peer support staff, and case managers. Moreover, an ongoing training module of 1.5 hours was incorporated into the New Employee Orientation to ensure that new hires were also well-prepared, facilitating their integration during live virtual training sessions.

#### 2.3.2 Patients

A total of 11,495 patients receiving MOUD were linked to the Recovery Connect app during the study period. All existing and new patients who connected via the app in the study period were included in the analysis.

### 2.4 Recovery connect app

The Recovery Connect app is a CMS branded version of Recovery Path, a digital tool designed to enhance and complement the delivery of MOUD. It provides a platform for patients and MHPs to interact, monitor progress, and access evidence-based resources to support ongoing recovery. MHPs and patients have separate versions of the app which they download separately and then connect via a QR code that is unique to each MHP.

For patients, the app offers several key features, including access to a library of over 200 coping skills, meditations, psychoeducational modules, and motivational enhancement resources to help manage cravings, triggers, and other challenges that may present in their daily lives. It also provides tools for self-monitoring cravings, triggers, motivation, anxiety, depression, sleep patterns, upcoming risky events, interpersonal issues, and relapse prevention planning. Additionally, it creates a secure and easy text-based communication channel between patients and their MHP, aiming to foster regular connection, trust, and relational accountability.

For MHPs, the Recovery Connect app provides visibility into patient progress, setbacks, and real-time data on factors such as cravings, mood, and substance use, allowing for timely identification and mitigation of high-risk situations. Historically, MHPs relied on time-intensive, phone-based patient outreach, which often had limited success in establishing contact. In contrast, the app streamlines communication and care coordination, seeking to enable more effective and efficient patient interactions. The app also allows for automated collection and scoring of patient-reported outcomes and treatment adherence data, facilitating measurement-based care and quality improvement efforts. It provides MHPs the ability to remotely suggest evidence-based coping strategies and psychoeducational modules tailored to individual patient needs. To reduce administrative burden on MHPs and improve documentation accuracy, the app was integrated with the CMS medical record system. This integration enables the automated creation of case notes that document MHPs’ time spent reviewing patient progress data, completing app-based care delivery tasks, and interacting with clients through the tool.

**Figure 1:**
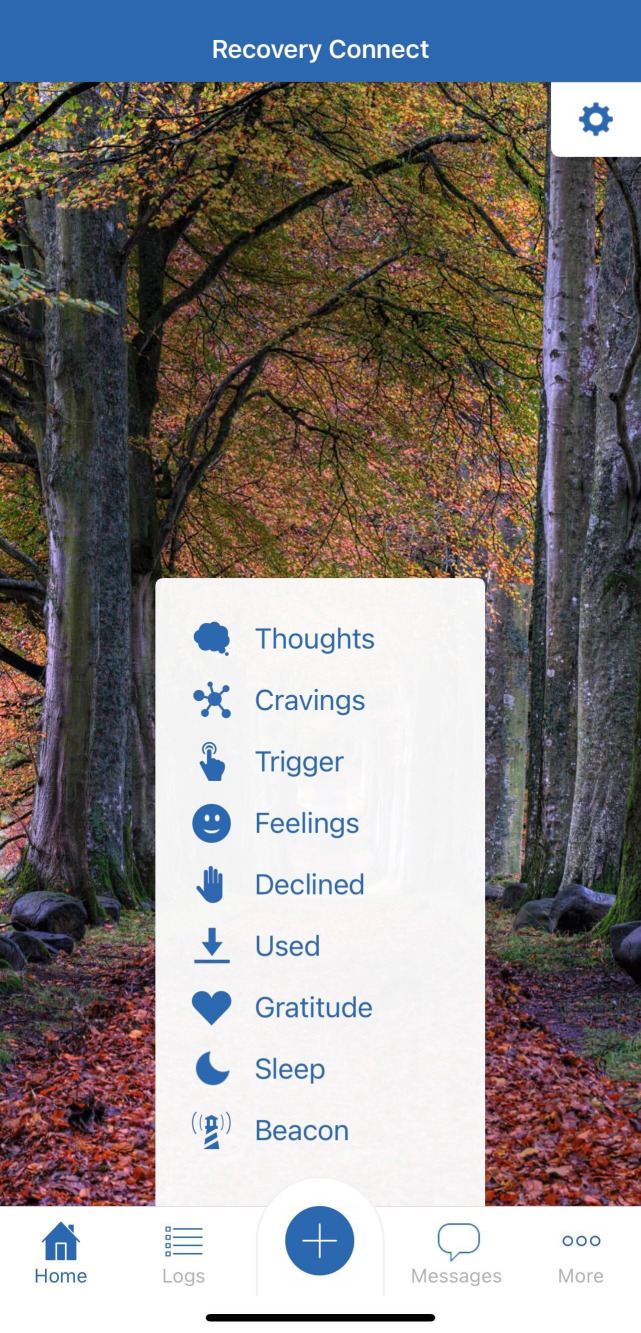
Main menu of RC app.

**Figure 2:**
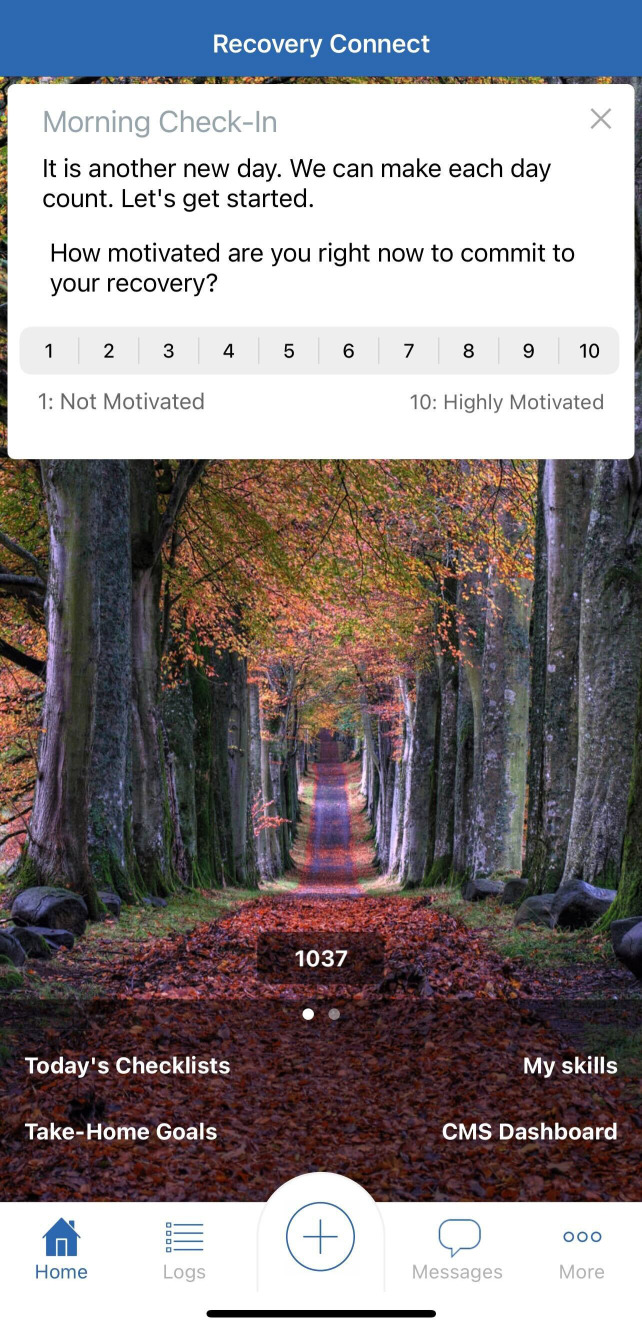
Example of RC daily check -in.

**Figure 3:**
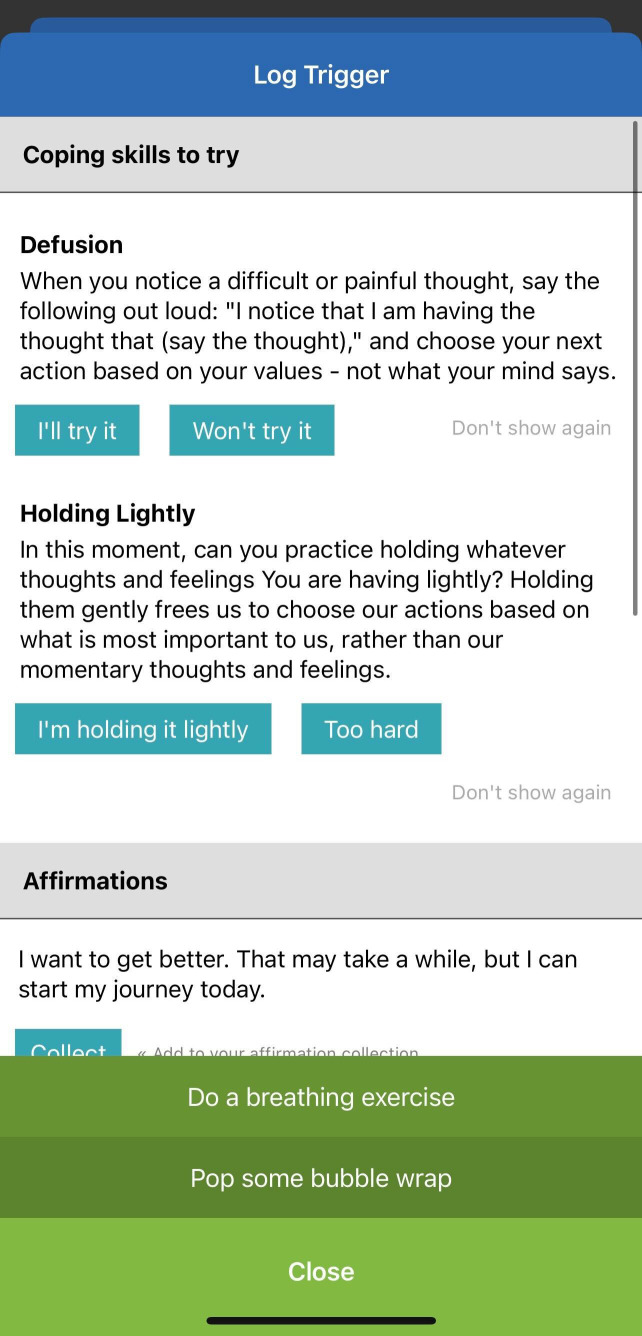
Example navigation of the RC app, including coping skills.

Overall, the Recovery Connect app aims to augment and scale high-quality MOUD care by leveraging digital technology to enhance patient engagement, provider-patient relationships, and access to evidence-based resources between clinic visits. Screenshots of different aspects of the app are shown in **Figures 1**, **2**, **3**.

### 2.5 Data collection

This study involved the collection and analysis of data related to the implementation and use of the Recovery Connect app within CMS’s OTP clinics during the 12-month period. The data collection process is summarized in the following sections:

#### 2.5.1 Acceptance measures

To assess the acceptance of the Recovery Connect app, early in the implementation, from July to September 2023, we provided optional questionnaires both to patients who linked on the app and the MHPs who were trained on use of the app. The instruments aimed to capture users’ perceptions, attitudes, and experiences related to the use of the Recovery Connect app within the MOUD program. Quantitative data from these questionnaires was explored thematically to understand the overall acceptability of the app as an adjunctive tool in OUD treatment.

Patient Questionnaires: Upon downloading and starting use of the app, patients were given a set of 18 optional questions, to be answered anonymously, around their intended use of the app and acceptance of digital tools and technology. These Likert-scale type questions, scored from 1-5, were adapted from previous questionnaires on technology acceptance and made up the following constructs: Intention to Use (ITU), adapted from 15; Perceived Usefulness (PU), adapted from 16; Perceived Ease of Use (PEOU), adapted from 16; Attitude (A), adapted from 17; Trust (T), adapted from 18; Technology Anxiety (TA), adapted from 18; Social Influence (SI), adapted from 19 and 20; and Perceived Health Threat (PHT), adapted from 17. Two open-ended questions were also included as part of the Usage Behavior (UB) construct. Responses to the two open-ended questions were analyzed independently by 2 researchers using thematic analysis, following the Braun and Clarke (21) framework. First, responses were familiarized through repeated reading, and initial codes were generated to capture key concepts. These codes were then grouped into potential themes, which were iteratively reviewed to ensure coherence and alignment with the research objectives. Themes were identified through a combination of inductive and deductive approaches, where emergent patterns were compared with constructs from the Technology Acceptance Model (TAM) framework. Codes and themes were then refined through iterative reflection and comparison, discrepancies resolved through discussion, and the final themes were categorized into facilitators (UB1) and barriers (UB2) toward app acceptance. While formal saturation was not assessed due to the structured survey format, thematic convergence was evident across responses, suggesting adequate coverage of dominant ideas. The full baseline questionnaire can be found in Appendix 1.

After 4-weeks, patients who had answered the initial survey were then sent an email to complete a follow-up survey with the same constructs included and questions amended to reflect their continued use of the app, rather than the initial use of the app.

MHP Questionnaire: MHPs trained on use of the Recovery Connect app during this period (July-September 2023) were invited to complete a survey which included a range of questions related to their current outreach methods, visibility into their patients’ progress in between visits, and expectations related to use of the app and the app’s potential to improve patient care. A similar thematic analysis was performed on the two open-ended questions for MHPs, which were also regarding what they would use RC for, and what, if any, were their primary concerns.

#### 2.5.2 Engagement measures

For every patient linking on the app, data on engagement, including metrics such as self-monitoring entries and number of messages sent to MHPs via the app were collected. This was deemed to be a more reliable way of measuring meaningful engagement, as opposed to simply opening the app, which can be misleading (an app can be opened and closed or just glanced at without the user actually gaining anything from that action). We focused on self-monitoring entries and clinician-initiated messaging because these actions represent active participation and relational accountability, both of which are clinically meaningful in OUD treatment. These metrics capture whether patients are consistently tracking their recovery process and whether clinicians are actively reaching out to support them. Additionally for each linked patient we collected the number of messages sent from their MHP via the app. Given the importance of the 30-day retention rates we also collected weekly usage data for the first 4 weeks after linking on the app to test for differences in this key period and their possible association to retention outcomes.

#### 2.5.3 Treatment retention analysis

A key outcome of interest in this study was the impact of the Recovery Connect app on patient retention in MOUD programs following admission or readmission, as such we undertook this analysis in the subset of new admissions/readmission patients who linked with their counselor via the app within the first 2 weeks of being admitted into the program.

The primary retention outcome measure (Days30) is defined as a patient not dropping out (i.e. begun a 30-day gap in treatment) within the first 30 days. As per federal guidelines, patients who abruptly leave the program can be readmitted within 30 days of departure without repeating the initial assessment procedure (22). Therefore, a patient is considered having ‘dropped out’ and routinely discharged if they have a 30-day gap in treatment, and the first day of that gap is recorded as the day they dropped out. In other words, if the last dose administered prior to discharge was less than 30 days after admission, the 30-day retention is operationalized as 0, and otherwise it is 1.

Comparative Analysis: To assess the potential impact of the Recovery Connect app on retention, 30-day retention rates observed during the study period were compared to historical retention data from all CMS clinics prior to the implementation of the Recovery Connect app. Additionally, a separate analysis examined 30-day retention data from clinics in Arizona, comparing outcomes before and after the implementation of Recovery Connect. Arizona was selected for this analysis as it was the first state in which the app was introduced, has the largest patient population, and the app had been implemented in all clinics within the state during the study period. This provided sufficient data for a pre-post analysis using the same group of clinics within the same region.

## 3 Results

### 3.1 Overall sample

During the 12-month study period from July 2023 to June 2024, a total of 11,495 patients receiving MOUD were linked to the Recovery Connect app across the 53 participating clinics. 7,895 (68.7%) were based in Arizona (24 clinics), 994 (8.6%) in Ohio (9 clinics), 991 (8.6%) in Texas (5 clinics), 842 (7.3%) in Wisconsin (5 clinics), 411 (3.6%) in Indiana (1 clinic), 187 (1.6%) in Colorado (4 clinic), 105 (0.9%) in Michigan (2 clinics), 53 (0.5%) in Minnesota (2 clinics), and 17 (0.1%) in Oregon (1 clinic). This reflects both the fact that most CMS clinics are based in Arizona, but also the gradual roll out of the app during the study period, which started in Arizona and proceeded across the other states mentioned above.

The mean age at admission of the patient cohort was 38.4 (SD 11.2), which reflects the population at CMS overall. The largest age groups were those aged 18-29 (20.2%) and aged 30-39 (41.7%). 61.6% of the patient cohort was of white ethnicity, and 50.4% identified as male. 8.1% had moderate/severe depression at admission, 12.4% had moderate/severe anxiety at admission, and 14.0% had unstable housing.

A majority of the cohort were in their first treatment episode (65.0%), 19.2% were in their second episode, 8.0% in their third, and 7.8% were in their 4th episode or more. A vast majority of patients were under methadone-only treatment (90.1%), whilst 7.9% were on buprenorphine.

### 3.2 Usage

Patients’ engagement on the app was assessed using the number of self-monitoring entries logged on the app, such as morning and evening solution-focused check-ins, behavior chain analyses of triggering events or cravings, and cognitive-behavioral thought processing. Patients included in this cohort had completed a total of 272,541 self-monitoring activities within the app during the study period, with a mean of 23.7 (95% CI: 22.0 - 25.4). Since the app has been implemented sequentially, we also operationalized usage variables to look at counts in the first 12 weeks after linking for all patients. The mean self-monitoring entries logged in the first 12 weeks after linking was 10.9 (95% CI: 10.2 - 11.5). This was significantly higher in patients in their first treatment episode (12.0; 95% CI: 11.2 - 12.7) than in those on their second or subsequent treatment episode (9.0, 95% 7.9 - 10.1) (two sample t-test < 0.001).

To evaluate patient-clinician communication through the app, the number of messages sent by patients to their MHPs and messages/comments received from MHPs was examined. Patients received a total of 417,840 messages from their MHPs, with a mean of 36.3 messages (95% CI: 35.4 - 37.3) and a mean of 12.4 (95% CI: 12.1 - 12.8) in the first 12 weeks. They also sent a total of 128,128 messages, with a mean of 11.1 (95% CI: 10.6 - 11.7), and a mean of 5.1 (95% CI: 4.9 - 5.3) in the first 12 weeks after linking. Those on their first treatment episode had significantly more messages received from MHPs (13.2; 95% CI: 12.7 - 13.6) in their first 12 weeks than those on their second or more treatment episodes (11.1; 95% CI: 10.6 - 11.6) (two sample t-test < 0.001). They also had sent more messages to MHPs in the first 4 weeks (5.6; 95% CI: 5.3 - 5.9) than those on their 2nd episode or more (4.2; 95% CI: 3.9 - 4.5) (two sample t-test < 0.01).

To further explore factors associated with app engagement (self-monitoring entries) in the first 12 weeks, a multiple linear regression analysis was conducted. Age (β = .10, SE = .04), gender (β = -5.81, SE = .98) and treatment episode (β = -1.36, SE = .19) were all found to be a significant predictor (p < 0.01) of overall app usage: therefore older patients, females and those with less treatment episodes were found to engage with the app significantly more. Ethnicity, having moderate/severe depression and anxiety at admission, as well as unstable housing, did not significantly predict engagement.

### 3.3 Acceptance of the app

#### 3.3.1 Early acceptance

828 patients completed the initial survey. Each response was scored on a scale of 1-5, where ‘Strongly Agree’ = 5 and ‘Strongly Disagree = ‘1’. Breakdown of responses can be seen in **Table 1**. The overall mean acceptance score was 4.08 [4.05 - 4.12] (for the negatively worded questions in the TA and PHT construct, the points were reversed for the purposes of this calculation). We conducted Krusk-Wallis tests for non-parametric data (since the scores were non-normally distributed) to explore the possible association with scores to clinic, device type (IoS, Android, tablet), and days in treatment, which were additional questions asked in the survey. There were no statistically significant associations between these variables and the overall score.

**Table 1:** Distribution of responses for each question in baseline patient survey.

Question	Total responses	Strongly disagree	SD %	Disagree	D %	Neutral	N %	Agree	A %	Strongly agree	SA %
question_ITU1	828	4	0.5	10	1.2	121	14.6	318	38.4	375	45.3
question_PU1	828	6	0.7	11	1.3	178	21.5	320	38.6	313	37.8
question_PU2	828	6	0.7	12	1.4	86	10.4	349	42.1	375	45.3
question_PU3	828	6	0.7	13	1.6	127	15.3	339	40.9	343	41.4
question_PEOU1	828	5	0.6	5	0.6	85	10.3	383	46.3	350	42.3
question_PEOU2	828	2	0.2	9	1.1	93	11.2	378	45.7	346	41.8
question_A1	828	4	0.5	14	1.7	105	12.7	359	43.4	346	41.8
question_A2	828	5	0.6	12	1.4	71	8.6	354	42.8	386	46.6
question_A3	828	8	1.0	14	1.7	87	10.5	339	40.9	380	45.9
question_T1	828	11	1.3	9	1.1	143	17.3	310	37.4	355	42.9
question_T2	828	5	0.6	6	0.7	134	16.2	335	40.5	348	42.0
question_TA1	828	319	38.5	262	31.6	101	12.2	71	8.6	75	9.1
question_TA2	828	291	35.1	265	32.0	143	17.3	66	8.0	63	7.6
question_SI1	828	7	0.8	26	3.1	318	38.4	281	33.9	196	23.7
question_SI2	828	12	1.4	34	4.1	292	35.3	305	36.8	185	22.3
question_SI3	828	3	0.4	7	0.8	176	21.3	339	40.9	303	36.6
question_PHT1	828	232	28.0	164	19.8	173	20.9	160	19.3	99	12.0
question_PHT2	828	419	50.6	225	27.2	99	12.0	46	5.6	39	4.7

The breakdown for each of the constructs is as follows (correlations with days in treatment are noted where there was a significant association, and there were no significant associations with clinic or device type in any construct):

#### 3.3.2 Intention to use

The mean acceptance score for ITU1 was 4.27 [4.21 - 4.32]. 83.7% Strongly agreed/Agreed with the statement ‘I intend to use the Recovery Connect app as part of my treatment’, with only 1.7% Disagreeing/Strongly disagreeing. A Spearman’s rank correlation test indicated a statistically significant, negative association between ITU1 scores and days in treatment (ρ = -0.150, p < 0.001), suggesting that there is more intent to use the app in those who are earlier in their course of treatment.

#### 3.3.3 Perceived usefulness

The mean acceptance score for the PU construct was 4.21 [4.16 - 4.26]. Those strongly agreeing/agreeing to the 3 questions in this construct ranged from 76.4 - 87.4%, whilst those disagreeing/strongly disagreeing ranged from 2.1 - 2.3%. Similar to intention to use, a Spearman’s rank correlation test indicated a statistically significant negative association between PU scores and days in treatment (ρ = -0.132, p < 0.001).

#### 3.3.4 Perceived ease of use

PEOU had a mean acceptance score of 4.28 [4.24 - 4.33]. 88.5% agreed or strongly agreed that the RC app would be clear and understandable, and 87.4% believed it would be easy to use. Only 1.2 and 1.3%, respectively, disagreed or strongly disagreed. A Spearman’s rank correlation test showed a statistically significant negative association between PEOU scores and days in treatment (ρ = -0.073, p = 0.035).

#### 3.3.5 Attitude

This construct had a mean acceptance score of 4.29 [4.24 - 4.34]. There was a 85.1 - 89.4% agreement (and 21. -2.7% disagreement) with questions related to comfort around tracking progress, sharing progress data, and sharing setbacks via the app. Again, a Spearman’s rank correlation test showed a statistically significant negative association between attitude scores and days in treatment (ρ = -0.120, p < 0.001).

#### 3.3.6 Trust

The mean acceptance score for the trust questions was 4.21 [4.16 - 4.26], and 80.3 - 82.5% agreed/strongly agreed that the app would protect their privacy, and the services were backed by professional expertise and evidence-based practices. Spearman’s rank indicated a slight statistically significant association between shorter time in treatment and higher trust scores (ρ = -0.131, p < 0.001).

#### 3.3.7 Technology anxiety

Mean acceptance score for this construct was 3.81 [3.73 - 3.88]. 70.2% did not agree/strongly disagreed with having concerns around use of the app due to lack of technical skills, and 67.1% did not agree/strongly disagreed with having concerns around internet connection issues preventing use of the app.

#### 3.3.8 Social influence

The mean acceptance score for the SI construct was 3.88 [3.83 - 3.93]. 57.6% and 59.2%, respectively, agreed/strongly agreed that family/friends/colleagues had full confidence in technological innovation and were open to trying new things. Only 4.0% and 5.6%, respectively disagreed or strongly disagreed with these statements. In terms of approval of use of the app by family/friends/colleagues, agreement was higher still at 77.5%, with only 1.2% disagreeing/strongly disagreeing.

#### 3.3.9 Perceived health threat

The mean PHT acceptance score was 3.73 [3.66 - 3.80]. 47.8% Strongly disagreed/disagreed about having concerns about their substance use, whilst 31.3% strongly agreed/agreed. 77.8% did not agree/strongly disagreed to having concerns about dropping out of treatment, whilst 10.3% did agree/strongly agree. There was a statistically significant, moderate, positive correlation between longer treatment duration and higher PHT scores, indicating that those in the earlier stages of their treatment had more concerns about their use/dropping out. Spearman’s rank (ρ = 0.219, p < 0.001).

#### 3.3.10 Usage behavior

This construct was analyzed thematically via 2 open-ended questions: What is the primary reason you believe you will use the Recovery Connect app for? (UB1) and What is your primary concern, if any, with using the Recovery Connect app? (UB2). These represented both the facilitators and barriers, respectively, toward acceptance with the app.

##### 3.3.10.1 Primary reason for using Recovery Connect (UB1)

The following facilitators to acceptance were identified (with example responses for each):

###### 3.3.10.1.1 Staying connected with MHPs

By far the most common theme, many patients expressed that the app would serve as a convenient and accessible means to communicate with their MHPs, who are referred to by patients as counselors. They highlighted the importance of having a direct line of contact, especially when in-person meetings were not possible or when they needed support outside of scheduled appointments. Patients appreciated the ability to send messages, ask questions, and receive timely responses from their MHPs through the app.

Examples:

“This is the best thing that has come my way to keep me connected to my counselor. I was starting to get a little frustrated. Sometimes it’s easier to write my feelings down than to express them in person.”

“To stay in contact with my counselor and to make sure to get things done off my checklist that I need to get done.”

“Keeping in contact with my counselor on a more personal and regular basis.”

###### 3.3.10.1.2 Tracking progress and maintaining accountability

Patients frequently mentioned using the app to track their recovery progress and maintain accountability. They believed that regularly logging their moods, triggers, and successes would help them stay mindful of their recovery journey. The app’s daily check-ins and self-monitoring features were seen as valuable tools for keeping themselves accountable and motivated.

Examples:

“I believe that consistency plays a big role in recovery. Since I have to get on the app every morning and evening to do check-ins that makes it consistent for me, which really helps in my recovery. This is the main reason I will use the recovery app.”

“Checking in and helping me stay on track and working towards my goals.”

“To track my recovery and to use the daily feelings log.”

###### 3.3.10.1.3 Accessing resources and coping strategies

Another common reason for using the app was to access helpful resources and coping strategies. Patients expressed interest in utilizing the app’s library of information, tools, and activities to support their recovery. They anticipated that the app would provide them with guidance, inspiration, and practical techniques to manage cravings, stress, and other challenges they might face.

Examples:

“Completing activities and games. Providing opportunities to learn new coping skills.”

“It’s another tool to retain more knowledge about recovery & how to better cope with triggers, urges, cravings, intrusive thoughts & anger.”

“Well I’m actually finding new things on it every time I use it & they all seem to be beneficial for my recovery!”

###### 3.3.10.1.4 Facilitating treatment engagement

Patients also viewed the app to enhance their overall treatment engagement. They believed that using the app would help them stay more involved in their recovery process, even on days when they couldn’t physically attend the clinic. The app’s features, such as appointment reminders and treatment plan tracking, were seen as ways to reinforce their commitment to recovery.

Examples:

“I think this app will help me be more involved in my treatment at CMS.”

“Keep track of my appointments and meetings.”

“Remembering to do things and being more effective on my treatment plan and personal and treatment goals because it all makes a difference on my overall mental health which affects my ability to be proactive in my treatment goals.”

###### 3.3.10.1.5 Seeking motivation and emotional support

Many patients expressed a desire to use the app as a source of motivation and emotional support. They hoped that the app’s encouraging messages, affirmations, and success stories would provide them with the inspiration and reassurance needed to maintain their sobriety. Patients also appreciated the idea of having a readily available support system through the app, especially during moments of vulnerability or when facing triggers.

Examples:

“For positive reinforcement and encouragement.”

“Insight and inspiration. Help me focus on my weak areas so I can make them stronger.”

“It gives me something to look forward every morning”

###### 3.3.10.1.6 Convenience and ease of use

Several patients mentioned that they would use the app because of its convenience and ease of use. They appreciated the accessibility of having the app on their phone, allowing them to engage with their recovery resources anytime and anywhere.

Examples:

“I love technology and I always have my phone within arm’s reach!!”

“Because it’s so accessible”

“I will use this app for its convenience. I like that it helps me organize my days better. I also enjoy being able to use the tools 24/7.”

##### 3.3.10.2 Concerns with using the Recovery Connect app (UB2)

Most respondents (69.6%) expressed no concerns regarding the use of the Recovery Connect app. Many simply stated “None” or “No concerns” in their responses.

Examples:

“I have no concerns about using the app!”

“I really don’t have any concerns about this app and to be honest I am very happy with it because it saves me so much time using the app so I don’t have to go to the clinic if I have an issue.”

“None - it’s perfect. I love using it.”

Of those that expressed some concerns, the following barriers to acceptance were identified (with example responses for each):

###### 3.3.10.2.1 Privacy and security

Some patients voiced concerns about the privacy and security of their personal information within the app. They mentioned potential data breaches, unauthorized access, or the sharing of sensitive information with unintended parties.

Examples:

“I suppose my main concern would be that the app itself is secure and safe. I wouldn’t want someone to breach the network and see my confidential information somehow.”

“My main concern is, will all my information stay confidential on this app? I don’t want whatever I talk about to be disclosed or leaked! I want to trust that this app will be used properly and abide by the HIPAA law.”

“Honestly how honest I can be for fear of not knowing fully who can see the information & what they can see. I’m trying to believe that it’s okay to be honest because this app will not benefit me if I’m not truthful.”

###### 3.3.10.2.2 Technical issues and app usability

A few patients expressed concerns about potential technical issues, such as app crashes, internet connectivity problems, or difficulties navigating the app’s features.

Examples:

“This app. could possibly have many “technical issues” that won’t get fixed or take long to be fixed!”

“Getting confused with the technical aspect of the app but I have my wife/caregiver to help me out.”

“Internet connection issues.”

###### 3.3.10.2.3 Time commitment and app engagement

Some patients were concerned about the time commitment required to effectively use the app and engage with its features consistently.

Examples:

“Will I actually take the time each day to use it.”

“I might not be able to check in every single day due to my responsibilities outside CMS.”

“That it will require too much of my already valuable time and attention.

###### 3.3.10.2.4 MHP responsiveness and communication

A few patients expressed concerns about a potential lack of responsiveness from their MHPs through the app, as well as the effectiveness of app-based communication.

Examples:

“That my counselor is actually going to get it and respond.”

“Not getting a response from my counselor.”

“Getting a reply to messages sent to my counselor during the same day, some messages could be urgent”

#### 3.3.11 Sustained acceptance

246 (29.7% of the 828 who answered the baseline survey) patients fully completed both the initial and follow-up survey 4 weeks later. The changes in mean scores for each construct are shown in **Table 2**.

**Table 2:** Baseline and follow-up acceptance scores for the subset of patients who completed both surveys (n = 246).

Construct	Initial survey mean (SD)	Follow up survey mean (SD)	Paired T-Test
ITU	4.25 (0.79)	4.26 (4.81)	0.828
PU	4.21 (0.70)	4.13 (0.75)	0.107
PEOU	4.30 (0.64)	4.41 (0.68)	0.018*
A	4.36 (0.66)	4.25 (0.73)	0.022*
T	4.23 (0.78)	4.23 (0.80)	0.964
TA	3.99 (1.03)	4.00 (1.05)	0.935
SI	3.88 (0.75)	3.88 (0.74)	0.874
PHT	3.80 (1.06)	3.82 (1.16)	0.725

Acceptance score remained high at 4 weeks for all constructs, with only Perceived Ease of Use and Attitude showing significant differences from baseline to follow-up. Linear regression analysis did not find any significant associations between changes in scores over time in these constructs and days in treatment prior to conducting the survey, suggesting changes in attitude and ease of use are independent of treatment duration prior to installing and using the app.

#### 3.3.12 MHP acceptance

88 MHPs (83.8% of the 105 who were invited to complete) responded to a survey delivered after initial training on use of the app. The survey contained questions regarding current client outreach methods and time spent, patient access to resources, technology acceptance, as well as use and expectations for the Recovery Connect app. The results from the individual questions are shown in **Table 3**.

**Table 3:** Full breakdown of Likert-scale MHPs survey responses.

Question	Strongly agree		Agree		Neither agree nor disagree		Disagree		Strongly disagree	
	n	%	n	%	n	%	n	%	n	%
Effective current outreach methods	4	4.7	32	37.2	32	37.2	16	18.6	2	2.3
Good visibility between visits	7	8.1	26	30.2	31	36.0	17	19.8	5	5.8
Good support outside of clinic	6	7.0	12	14.0	30	34.9	27	31.4	11	12.8
Technology makes me nervous	1	1.2	19	22.6	26	31.0	32	38.1	6	7.1
Recovery Connect:	3	3.5	7	8.1	13	15.1	33	38.4	30	34.9
Is easy to use	36	41.9	36	41.9	11	12.8	2	2.3	1	1.2
Promotes good clinical practice	32	37.6	41	48.2	7	8.2	2	2.4	3	3.5
Improves clinical performance	31	36.5	43	50.6	6	7.1	2	2.4	3	3.5
Improves assessment of clients’ progress	30	35.3	45	52.9	7	8.2	1	1.2	2	2.4
Makes time with clients more efficient	30	35.3	46	54.1	7	8.2	1	1.2	1	1.2
I have the intention to use RC	36	42.4	35	41.2	7	8.2	1	1.2	6	7.1

##### 3.3.12.1 Current outreach and patient access to resources

94% of MHPs had used a phone to reach their clients, 58% had used messages sent through the medical record, 52% had used email, and 35% had used text messages. On average, MHPs said that when trying to reach clients by phone, they were successful 33% of the time, and only 41.9% agreed or strongly agreed that their current outreach methods were effective. Furthermore, 38.4% agreed or strongly agreed that they had good visibility into a patient’s progress, 20.9% said they can easily extend support outside of clinic visits and 23.8% said that clients can access the resources they need when they need them.

##### 3.3.12.2 Acceptability of technology and the Recovery Connect App

Most of the MHPs who responded to the survey stated technology did not make them nervous, with only 11.6% agreeing or strongly agreeing with that statement.

For all questions relating to usability and acceptability of the app, there was a positive response in over 80% of MHPs surveyed. Questions were regarding ease of use, promotion of good clinical practice, improvement of clinical performance, assessment of progress, getting the most out of time with clients, and intent to use. The range of strong agreement/agreement for these was 83.5 - 89.4%, with only 2.4% - 8.2% disagreeing with those statements.

##### 3.3.12.3 Qualitative questions and thematic analysis

Two open-ended questions were asked in the MHP survey: “What is the primary reason you believe you will use Recovery Connect for?” and “What is your primary concern about using Recovery Connect in your work?”.

When asked what the primary reason for using Recovery Connect would be, MHPs revealed several reasons, centered around enhancing communication, increasing client engagement, and improving treatment effectiveness.

###### 3.3.12.3.1 Enhancing communication and connection with clients

A predominant theme was the desire to improve communication with clients. MHPs expressed that the app would help them “stay connected with my clients and provide encouragement” and “have more communication with my clients.” The app facilitates direct messaging and real-time communication, allowing MHPs to “keep in contact with my patients” and “connect with clients who don’t necessarily have time to be in the clinic for a talk.” By offering another mode of communication, the app helps in “creating more connection and being an active part of client recovery.”

###### 3.3.12.3.2 Increasing client engagement

MHPs highlighted the app’s potential to boost client engagement. They mentioned using it for “weekly engagement, check-in, progress monitoring, [and to] build rapport.” The app enables “engagements and homework,” allowing clients to participate actively in their recovery process. One MHP noted, “I will be able to engage with clients more,” emphasizing the app’s role in fostering continuous involvement. Another said the app “will help and be a great motivator for clients.”

###### 3.3.12.3.3 Facilitating check-ins and monitoring

The app serves as a tool for efficient check-ins and monitoring of clients’ progress, allowing MHPs to keep track of clients’ well-being between sessions. MHPs planned to use it for “quick check-ins,” “appointment reminders/follow-ups, session discussion topics/tracking,” and to “identify ones who are engaged in their treatment.”

###### 3.3.12.3.4 Addressing barriers to treatment

MHPs recognized the app’s role in eliminating treatment barriers and appreciated the app’s convenience for both themselves and their clients. It accommodates clients who are “more phone/text oriented” and “on the go.” It was deemed to be particularly useful for “clients who do not like face to face” interactions or “do not answer calls” One MHP mentioned, “I am only on site to see my caseload in person twice a week, so using this app will be another way I can stay in contact more and reply to my clients faster if need be”

###### 3.3.12.3.5 Building relationships and rapport

MHPs expressed a desire to “develop relationships”, “build rapport” and “gain rapport and trust a lot more within a less serious way.”

###### 3.3.12.3.6 Real-time updates and insights

The ability to receive real-time updates on clients’ progress was another key reason for using the app. MHPs valued that it “will give me the real-time updates and follow up with my clients” and allows them to “better understand their needs.”

###### 3.3.12.3.7 Time efficiency

Some MHPs noted the app as a “time saver,” providing “more in-depth insight” and helping them “get more counseling time.”

When asked about their primary concerns, several themes emerged, primarily technology-related challenges:

###### 3.3.12.3.8 Clients’ access to technology

MHPs mentioned that clients might lack the necessary devices or internet access to use the app. One mentioned was concerned with “My clients that don’t have access to a smartphone” whilst another said “There will be some barriers with client being able to access the app due to the preexisting cellphone barriers that the clients already have.”

###### 3.3.12.3.9 Technological proficiency

Concerns were raised about both personal apprehension in regarding new technology (“I’m not good with technology” or “I sometimes have difficulty with technology”) and clients’ ability to navigate the app (“Patient not being technology savvy”, “The clients figuring out how to use it or getting the client to participate” or “Clients figuring the app out”).

###### 3.3.12.3.10 Client engagement and participation

Another concern was that clients might be unwilling or unmotivated to download or consistently use the app, thus reducing its intended impact. Some examples include: “My clients just won’t use it”, “The clients not wanting to download and use the service”, “Client resistance” and “Clients not taking advantage of this great app!”. In addition, some were worried that clients might misuse the app or misunderstand its purpose. One clinician mentioned “Concerns with inappropriate messages/images being sent” and another “That patients will assume this takes the place of counseling”.

###### 3.3.12.3.11 Time constraints and workload

The integration of the app was seen by some MHPs as adding to their already demanding schedules, raising concerns about time management and burnout. Some examples include: “Extra work”, “Additional requirements for overworked counselors [MHPs]”, and “Having the time between appts and documenting to use the app”. Furthermore, MHPs expressed that incorporating the app might detract from client interactions during sessions or overwhelm them during busy periods (“Not enough time during an intake to be able to use it effectively”, “Distractions and being misunderstood by client due to hurriedness of clinic”).

###### 3.3.12.3.12 Privacy and confidentiality concerns

Although this was not cited by more than a handful of MHPs, one did mention that “I will not put client PHI on my personal phone. I will need to ensure that I am remembering to check on client status on the desktop in office.” Another simply said “Client Confidentiality” as a concern and yet another mentioned “Not being able to have a good documentation”.

###### 3.3.12.3.13 Boundary setting

There was some apprehension that the app could lead to clients expecting constant availability, potentially intruding on MHPs personal time. Examples on this theme include: “Neediness on the part of clients”, “Too much availability” and “We just have to make sure they know this is not for after hours”.

###### 3.3.12.3.14 No concerns

Many MHPs expressed no concerns, with some taking the time to share their optimism surrounding the app, with responses such as “No concerns at this time, very excited about using the app”, “This app was a great idea” and “This is a life changer and a game changer”.

### 3.4 Retention analysis

1,957 patients in the sample were eligible for retention analysis. A larger majority of this subsample (1,498, 76.5%) compared to the overall sample comes from clinics in Arizona, since these were the first to roll out the app. The mean age at admission of this subsample was 33.4 (SD 15.5), 53.3% was of white ethnicity, and 47.4% identified as male. 56.6% were in their first treatment episode, 20.3% in their second, 9.9% in their third, and 13.3% in their fourth episode or more.

Overall retention (Days30) in this subsample was 78.0% (95% CI: 76.2 - 79.9). For Arizona clinics only, retention was 76.2%. For comparison, retention in patients from all clinics who did not have access to the app in January-June 2023 was 65.8%. In Arizona, retention in that same time period was 63%.

The mean number of daily doses within the first 30 days post admission in this subsample was 22.6 (95% CI: 22.3 - 23.0). In Arizona, it was 22.1 (95% CI: 21.6 - 22.5). For comparison, across CMS in January-June 2023 the mean was 17.8 and 30-day mean daily doses in Arizona for that same time period was 17.9.

A multivariable logistic regression analysis was conducted to examine the associations between baseline demographics, clinical characteristics, early usage and 30-day retention. Predictor variables included age, ethnicity, gender, presence of moderate depression, moderate anxiety, and unstable housing at baseline. We also included early patient engagement, via number of self-monitoring entries and messages sent to MHPs done in the first 2 weeks, and the presence of an engaged MHP. This last variable was dichotomised as having been sent 2 or less, or at least 3 messages from their assigned MHP in the first 2 weeks. The cutoff of 3 messages was chosen pragmatically to reflect what our clinical teams identified as a meaningful minimum threshold for proactive clinician engagement. Self-monitoring entries in the first 2 weeks (OR = 1.04, 95% CI: 1.01 - 1.06, p < 0.01), an engaged MHP (OR = 1.42, 95% CI: 1.07 - 1.90, p = 0.01), and less treatment episodes (OR = 0.89, 95% CI: 0.83 - 0.95, p < 0.01) were significantly associated with 30-day retention. Messages sent to counselors from patients was not significantly associated with 30-day retention Therefore, for every unit increase in self-monitoring entries, the odds of a patient being retained in treatment increased by 4% and having a MHP who sends more than 1 message a week during the first 2 weeks is positively associated with an increased likelihood of retention.

## 4 Discussion

The integration of the Recovery Connect app into MOUD programs across 53 clinics over a 12-month period yielded insights into the acceptability, engagement, and initial clinical outcomes associated with digital health interventions in OUD treatment. The observational nature of the study allowed for the examination of real-world patterns of app adoption, usage, and its integration within existing MOUD workflows, without experimental manipulation.

### 4.1 Main findings

#### 4.1.1 High patient acceptance

Initial acceptance of the app was high. Mean acceptance score was 4.08 out of 5, indicating strong agreement with positive statements around the app’s usefulness, ease of use, and intention to use it. Specifically, 83.7% agreed or strongly agreed with the statement ‘I intend to use the Recovery Connect app as part of my treatment’, with only 1.7% disagreeing. The significant negative correlation between acceptance scores and days in treatment suggests that introducing digital interventions at the outset of treatment may maximize patient engagement, whilst also suggesting that patients who are longer in treatment and more likely to be ‘stable’ may see less initial benefit of an app that supports their treatment.

#### 4.1.2 Engagement patterns

Patients showed positive engagement with the app, and by observing self-monitoring entries completed we were able to quantify meaningful engagement, i.e. not ‘just’ opening and browsing the content but completing tasks in the app which form part of the core functionalities. Engagement was higher in the first 4 weeks amongst patients who were in their first treatment episode. Age, gender and ethnicity were also predictors of engagement. This is in line with previous findings showing non-white ethnic groups as being less likely to use digital health technologies (23), females as more likely to engage (24), and older adults engaging more with digital platforms than their younger counterparts (25), However, there is yet no consensus on these patterns of engagement across demographic groups, and its important to consider individual characteristics of the app and its users to maximize engagement (26).

#### 4.3.3 Clinician-patient interactions through the app

Enhanced communication is vital in OUD treatment, as it fosters therapeutic alliance and provides timely support when needed. Prior studies have shown effectiveness with videoconferencing (27) and mobile apps (28) in increasing communication. Further, a recent systematic review identified the importance of linking apps to providers and how clinician support helps overcome tech literacy, whilst enhancing self-monitoring and engagement with the app (29). In our study we saw both a high rate of messages sent to MHPs from patients, and messages and comments sent from MHPs to their patients. Since the messages sent from MHPs to patients were also a predictor of increased retention rates, emphasizing the critical role of enhanced communication via the app is key in training and implementation of digital tools such as Recovery Connect.

#### 4.3.4 High clinician acceptance

Clinician acceptance of the app was also high. Our survey indicated that clinicians recognized the potential of Recovery Connect to assess clients’ progress, make them more time efficient, promote good clinical practice and subsequently improve their performance in client care. In line with the suggested need for alternative communication channels, it seemed evident that the app was filling a gap in their care and clinicians welcomed use of the app to enhance said care.

Notably, the theme on communication and increased contact between sessions was the one most cited by clinicians in the qualitative question on primary reason for using the app. Engagement with clients seemed to emphasize the app’s role in fostering continuous involvement from both clinicians and their clients. The check-ins, appointment reminders and coping skills, being accessible any time of day, was also cited, as support of the recovery journey outside of scheduled appointments seemed an important feature for clinicians. In addition, the app showed promise in overcoming logistical challenges that act as barriers to treatment, allowing clinicians to stay in contact more and respond faster when needed. Overall, the increase in communication between sessions has the potential to help build a stronger therapeutic relationship, building accountability on both sides and leading to higher retention in treatment. Prior research on video conferencing as an added component of on-site visits in MOUD programs discuss the importance of an expanded continuum of care and facilitation of attendance and retention through digital interventions (27).

#### 4.3.5 Positive impact on retention rates

A key finding of this study was the improvement in 30-day retention rates among patients who used the app. Using comparable historical data, 30-day retention rose by 12.2% overall and 13.2% in Arizona. Significant predictors of increased retention were the number of self-monitoring entries logged by patients, and messages received from MHPs in the first 4 weeks after linking on the app. These findings underscore the critical role of patient engagement and clinician support in enhancing retention. Early retention is particularly important in MOUD programs, as dropout during the initial stages is associated with poorer long-term outcomes (30). The ability of the app to facilitate self-monitoring and enhance patient-clinician communication may contribute to sustained engagement in treatment. 30-day dosing rates were also high, and these may also be an indicator of long-term retention, which would be worthwhile to confirm in future studies.

### 4.2 Strengths and limitations of the study

This study has several notable strengths. The Recovery Connect app represents an innovative intervention designed to address key barriers in Medication for Opioid Use Disorder (MOUD) programs by incorporating features such as real-time monitoring, tailored evidence-based resources, and enhanced communication capabilities. The Recovery Connect app was designed to complement rather than disrupt existing MOUD workflows. Through the integration with standard clinic operations, it minimized the burden on staff and facilitated adoption, an essential factor in real-world implementation. The study itself included a large and diverse sample of nearly 10,000 patients from 49 clinics across nine states, showing its acceptability and feasibility across different demographic and geographic contexts. The evaluation of app usage through measures like self-monitoring entries, patient-to-clinician messages, and clinician-to-patient messages, provided a more nuanced picture of patient engagement. These metrics allow for a richer understanding of how the app facilitated meaningful interactions, and their effect on clinical outcomes, rather than relying on simplistic usage statistics. The study’s qualitative analysis provided a detailed account of the facilitators and barriers to app use for both patients and clinicians. This information provides actionable insights for refining the app and informing future implementations. By identifying the specific needs of users, the study lays the groundwork for improving training and technical support to maximize adoption and impact in future.

The study also has limitations worth noting. The lack of a control group and observational nature of the study certainly prevents from drawing definitive causal conclusions about the app’s impact on retention and engagement with services. Indeed, changes in retention rates and engagement could be influenced by external factors not accounted for, such as policy changes, staffing patterns, or broader trends in MOUD practices during the study period. Additionally, there is potential selection bias to consider, as patients and clinicians who chose to use the app could inherently be more motivated to engage in their treatment, and these individuals would have a higher likelihood of recovering. This limitation underscores the importance of including concurrent non-user groups or randomized allocation in future evaluations, which would allow for clearer assessment of whether outcomes differ systematically between app users and non-users at baseline. Furthermore, while the study focused on early retention, it does not capture long-term outcomes such as sustained recovery or relapse rates, leaving gaps in understanding the app’s impact over time. Long-term follow-up would also allow for the elimination of a potential Hawthorne effect, as the novelty of the app and the attention given to its implementation (for example extra training sessions and surveys) might have temporarily increased engagement and retention.

### 4.3 Implications for future research, practice and policy makers

To build on the results from this study, future research should focus on rigorous efficacy studies using randomized controlled designs, such as stepped-wedge trials, to establish causal links between app use and improved retention. Long-term follow-up studies are also needed to examine the app’s effects on sustained retention and relapse prevention. Additionally, understanding how specific types of engagement, such as the frequency and content of self-monitoring entries, influence outcomes would provide valuable insights into optimizing app design and use. Investigating variations in clinician acceptance and engagement and their impact on patient retention could further elucidate the app’s mechanisms of action. Broader outcome metrics, such as staff productivity and turnover, also merit further exploration. Finally, it is important to further explore differences between certain subgroups to be able to adapt this app and other similar technologies to maximize their impact regardless of sociodemographic or clinical characteristics.

The dominant qualitative themes also highlight practical directions for refinement: for example, patients’ emphasis on communication and accountability underscores the importance of strengthening real-time messaging and self-monitoring features, while clinicians’ concerns around workload and boundary-setting suggest that future training should emphasize efficient integration into workflows and strategies for managing client expectations.

From a clinical perspective, targeted training programs could be developed to support MHPs with lower levels of technology acceptance. Furthermore, the real-time data generated by the app may be used to tailor interventions to individual patient needs, ensuring a personalized approach that enhances treatment efficacy. It is important to note that these findings emerged from a large, multi-state provider network with substantial infrastructure and dedicated training resources. Smaller clinics or under-resourced programs may face distinct implementation barriers, including fewer staff, less technical support, and differing patient demographics. Translation of these findings to such contexts will require adaptation of training models, workflow integration, and support mechanisms to ensure feasibility and sustainability.

Policy makers should consider incentivizing the integration of digital tools like the Recovery Connect app into MOUD programs. Funding mechanisms to support the adoption of these tools and the infrastructure required for their implementation, including training and technical support, are critical. Additionally, standardized data collection and reporting practices for digital health tools would facilitate cross-program comparisons, enabling more robust evaluations of their impact and scalability.

## 5 Conclusions

The findings from this study demonstrate the feasibility of the Recovery Connect app to enhance MOUD programs by addressing barriers to retention and engagement through features like real-time monitoring, patient-clinician communication, and evidence-based resources, which foster accountability and support recovery. Engagement metrics such as self-monitoring entries and clinician-patient messaging may be key drivers of improved retention. Future research should explore long-term retention, engagement strategies, and scalability to fully realize the app’s potential to improve clinical outcomes. With thoughtful integration into clinical practice and policy, digital technologies such as the Recovery Connect app hold the potential to transform addiction treatment and improve the lives of individuals living with opioid use disorder.

## Data Availability

The dataset used in this study is not publicly available, however, a deidentified version of the dataset may be made available upon reasonable request from qualified researchers, subject to approval from CMS and the software developers. Requests to access these datasets should be directed to jorge.palacios@brighttherapeutics.com.
